# Glucosuria Interferes With Measurement of Effective Renal Plasma Flow Using *para*-Aminohippuric Acid, With a Focus on SGLT2 Inhibitors

**DOI:** 10.1016/j.ekir.2020.09.002

**Published:** 2020-09-06

**Authors:** Rosalie A. Scholtes, Isabel T.N. Nguyen, Erik J.M. van Bommel, Nel Willekes, Melanie van Nieuwenhuijzen, Daniël H. van Raalte, Jaap A. Joles

**Affiliations:** 1 Diabetes Center, Department of Internal Medicine, Amsterdam University Medical Center, VU University Medical Center, Amsterdam, the Netherlands; 2 Department of Nephrology and Hypertension, University Medical Center, Utrecht, the Netherlands

Effective renal plasma flow (ERPF) is defined as the total plasma volume delivered to all nephrons per time unit. The gold standard nonradioactive method to measure ERPF is by urinary clearance of *para*-aminohippuric acid (PAH),^1^ since, in low doses, PAH is almost completely cleared from the circulation during a single pass through the kidneys, with an extraction ratio of about 0.92.^2^ Therefore, PAH can be used to measure ERPF in mechanistic renal physiology studies. The attention for such trials has recently increased because of the introduction of the glucose-lowering drug class of sodium glucose cotransporter 2 (SGLT2) inhibitors.

SGLT2 inhibitors lower plasma glucose levels through blocking glucose reabsorption in the proximal tubule, in turn inducing glucosuria. These agents have received much attention because of their robust renoprotective effects in people with type 2 diabetes, which are thought to primarily rely on amelioration of increased glomerular pressure by modulation of kidney hemodynamic function due to activation of tubuloglomerular feedback.^3^ As such, Cherney *et al.*^4^ investigated the effect of SLGT2 inhibition on kidney hemodynamic function in patients with hyperfiltering type 1 diabetes and found a decrease in inulin-measured glomerular filtration rate and PAH-derived ERPF, which indicates afferent arteriolar vasoconstriction. In people with type 2 diabetes, we similarly observed a reduction of inulin-measured glomerular filtration rate; however, as ERPF was preserved, our data indicated that SGLT2 inhibition induced efferent arteriolar vasodilation.^5^

In our trial,^5^ we measured ERPF by urinary PAH clearance. We calculated fractional PAH extraction ratios based on timed-urine sampling and infusion rates to validate our method. Surprisingly, we initially measured low mean extraction ratios of PAH (Table 1). This occurred during clamped hyperglycemia (extraction ratio 0.65), but also during clamped euglycemia following treatment with the SGLT2 inhibitor dapagliflozin (extraction ratio 0.71). At these low extraction ratios, ERPF is drastically underestimated. We conducted a literature search regarding this phenomenon and found that hyperglycemic, i.e., glucosuric, conditions were shown to reduce urinary PAH.^6^, ^7^, ^8^ Whereas this was initially thought to be due to physiological competition between renal tubular glucose reabsorption and PAH secretion,^6^ it was later shown that PAH and glucose react in acidic urine samples, a process that is ongoing during storage at –20 °C. It is presumed that a Schiff base is formed between the *para*-amino group of PAH and the aldehyde group of glucose, which would occlude the amine group and reduce measurable free PAH.^6^ Premixing urine samples with NaOH to provide alkaline conditions (pH 9) totally prevents this reaction, as alkaline hydrolysis of the complex does not take place. However, alkalinization of the urine after the PAH-glucose reaction has occurred will not reverse the process. On the other hand, adding 1.2 N hydrochloric acid (HCl) afterward restores free PAH by reducing the availability of the nucleophile and allowing the back-reaction to predominate. Both methods enable correct measurement of urinary PAH concentrations independent of storage time.^6^

**Table 1:** Effect of glycosuria on renal hemodynamics before and after treatment with HCL in human and animal data

Human data	Hyperglycemic clamp at baseline (n = 44)			Euglycemic clamp after 12 wk dapagliflozin (n = 24)		
	Before HCl	After HCl	*P* value	Before HCl	After HCl	*P* value
PAH extraction ratio	0.65 ± 0.12	0.86 ± 0.09	**<0.001**	0.71 ± 0.15	0.88 ± 0.12	**<0.001**
Urinary PAH concentration, mmol/l	2.73 ± 0.83	3.52 ± 1.21	**<0.001**	2.41 ± 0.69	3.07 ± 0.94	**<0.001**
Effective renal plasma flow, ml/min	371 ± 111	488 ± 116	**<0.001**	389 ± 129	477 ± 121	**<0.001**
Urinary glucose levels, mmol/l	72 ± 50			33 ± 40		
Plasma glucose levels, mmol/l	19 ± 2			6 ± 0.5		

Animal data	Hyperglycemic rats (n = 20)					
	Before HCl	After HCl	*P* value			
PAH extraction ratio	0.24 ± 0.08	0.35 ± 0.10	**<0.001**			
Urinary PAH concentration, mmol/l	7.93 ± 4.60	11.1 ± 5.80	**<0.001**			
Urinary glucose levels, mmol/l	275 ± 183					
Plasma glucose levels, mmol/l	19 ± 6					

HCL, hydrochloric acid; PAH, *para*-aminohippuric acid.

Data are presented as mean ± SD. In all human cases, a paired sample *t* test was used to compare groups. In the animal samples, a Wilcoxon signed rank test for related samples was used because of skewed distribution of the variables despite log transformation.

Bold indicates statistical significance (*P* < 0.05).

We remeasured our urine samples after treatment with HCl and indeed found higher PAH extraction ratios in the expected range (Table 1). These HCl-treated urine samples were used for the final analyses in our previous manuscript.^5^ In line with previous literature,^6^ a negative correlation was found between storage time and PAH extraction ratio in people treated with dapagliflozin during a euglycemic clamp before treatment with HCl (Figure 1a) and this correlation disappeared after adding HCl to the urine prior to analysis (Figure 1b). During the hyperglycemic clamp at baseline, where urinary glucose levels were substantially higher, a similar phenomenon was observed, although this correlation did not reach significance. We speculate that the influence of storage time is absent in these samples because, as a result of the high glucose concentrations, the reaction between urinary PAH and glucose was (almost) completed when the samples were first measured.

**Figure 1:**
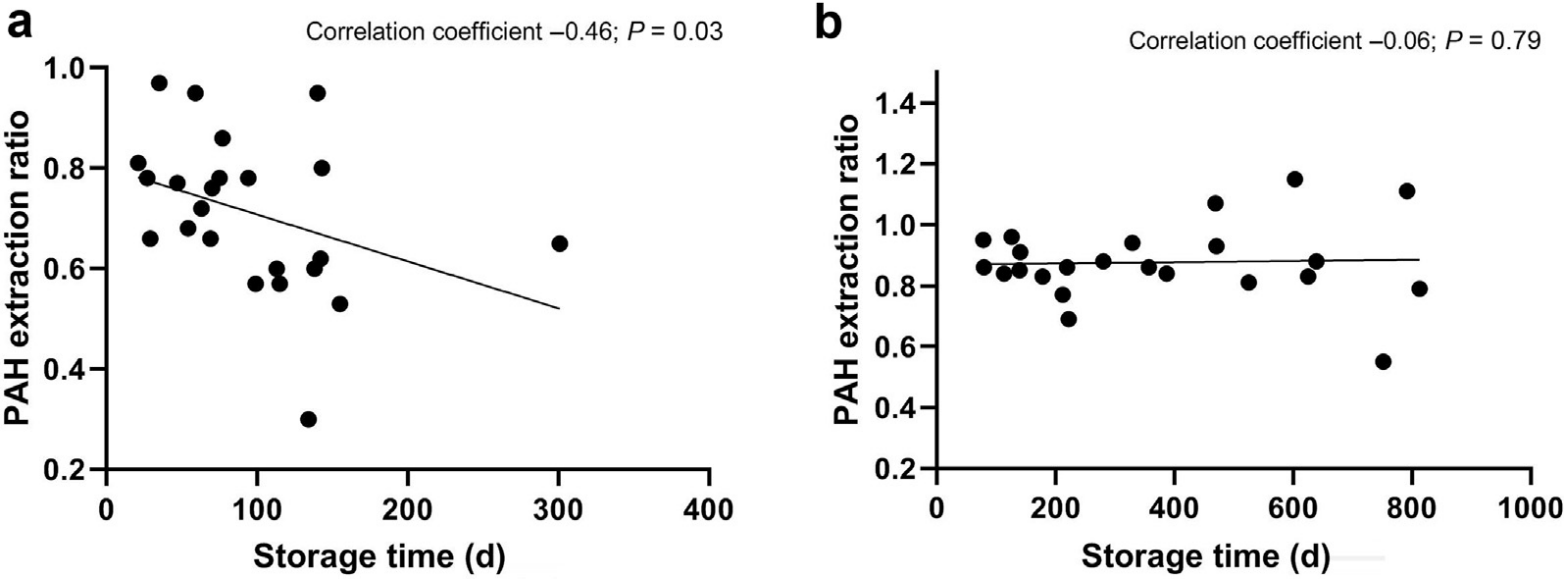
Correlation between storage time and *para*-aminohippuric acid (PAH) extraction ratio in people treated with dapagliflozin (a) before and (b) after treatment with hydrochloric acid during clamped euglycemia (n = 22).

In addition to these human data, we observed comparable results in male obese ZSF1 rats with hyperglycemia.^9^^,^S1 In these markedly hyperglycemic rats with severe glucosuria, extremely low extraction ratios were found. Urine PAH and the extraction ratio increased significantly after treatment with HCl (Table 1). Note that despite the increase in recovery of PAH after acidification, the extraction ratios of PAH were still very low in these rats with type 2 diabetes. This might be explained by the fact that extremely high blood glucose levels inhibit PAH secretion in these rats.S2 Hence, ERPF cannot be reliably estimated by PAH clearance in male obese ZSF1 rats.

Taken together, PAH-derived ERPF is increasingly being used in parallel with the revived interest in kidney hemodynamic function due to the development of SGLT2 inhibitors. Urinary clearance of PAH with timed-urine sampling is the most precise way to measure ERPF. However, researchers should be aware that even under euglycemic conditions, SGLT2 inhibitors induce glucosuria that interferes with accurate ERPF measurement as a result of glycosylation of PAH in stored urine samples. Pretreatment of urine with NaOH or adding HCl prior to measurement will overcome this problem.

## Disclosure

DHvR has acted as a consultant and received honoraria from Boehringer Ingelheim & Eli Lilly and Company Diabetes Alliance, Merck, Novo Nordisk, Sanofi, and AstraZeneca and has received research operating funds from Boehringer Ingelheim & Eli Lilly and Company Diabetes Alliance, AstraZeneca, and Novo Nordisk; all honoraria are paid to his employer. All the other authors declared no competing interests.

## Supplementary Material

Supplementary File (Word)
