# Supplementary material for: Glucosuria Interferes With Measurement of Effective Renal Plasma Flow Using para-Aminohippuric Acid, With a Focus on SGLT2 Inhibitors
Source: Kidney Int Rep. 2020 Sep 6;5(11):2052–4. doi: 10.1016/j.ekir.2020.09.002 (PMC7609950; doi:10.1016/j.ekir.2020.09.002)
Supplement: Supplementary File (Word) [file mmc1.docx]

Supplementary References

S1. Nguyen ITN, Brandt MM, van de Wouw J, et al. Both male and female obese ZSF1 rats develop cardiac dysfunction in obesity-induced heart failure with preserved ejection fraction. *PLoS One.* 2020;15:e0232399.

S2. Lote CJ, McVicar AJ, Yardley CP. Renal extraction and clearance of *p*-aminohippurate during saline and dextrose infusion in the rat. *J Physiol.* 1985;363:303–313.
